# Supplementary material for: Geospatial distribution and predictive modeling of onchocerciasis in Ogun State, Nigeria
Source: PLoS One. 2023 Mar 1;18(3):e0281624. doi: 10.1371/journal.pone.0281624 (PMC9977021; doi:10.1371/journal.pone.0281624)
Supplement: Attachment — Submitted filename: Response to Reviewers.docx [file pone.0281624.s002.docx]

. Please ensure that your manuscript meets PLOS ONE's style requirements, including those for file naming. The PLOS ONE style templates can be found at 
[https://journals.plos.org/plosone/s/file?id=wjVg/PLOSOne_formatting_sample_main_body.pdf](https://journals.plos.org/plosone/s/file?id=wjVg/PLOSOne_formatting_sample_main_body.pdf) and 
[https://journals.plos.org/plosone/s/file?id=ba62/PLOSOne_formatting_sample_title_authors_affiliations.pdf](https://journals.plos.org/plosone/s/file?id=ba62/PLOSOne_formatting_sample_title_authors_affiliations.pdf)

**2. We noticed you have some minor occurrence of overlapping text with the following previous publication(s), which needs to be addressed:**

**- [https://www.medrxiv.org/content/10.1101/2022.01.10.22269016v1.full](https://www.medrxiv.org/content/10.1101/2022.01.10.22269016v1.full)**

Response: This has been adjusted and highlighted within the text (See lines 407-409 and 459-462)

**In your revision ensure you cite all your sources (including your own works), and quote or rephrase any duplicated text outside the methods section. Further consideration is dependent on these concerns being addressed.**

Response: This has been addressed, all sources have been cited within the text including my own works

3. **Please provide additional details regarding participant consent. In the ethics statement in the Methods and online submission information, please ensure that you have specified what type you obtained (for instance, written or verbal, and if verbal, how it was documented and witnessed). If your study included minors, state whether you obtained consent from parents or guardians. If the need for consent was waived by the ethics committee, please include this information**.

Response: This was previously included in the manuscript (Line 199-208).

**4. In your Data Availability statement, you have not specified where the minimal data set underlying the results described in your manuscript can be found. PLOS defines a study's minimal data set as the underlying data used to reach the conclusions drawn in the manuscript and any additional data required to replicate the reported study findings in their entirety. All PLOS journals require that the minimal data set be made fully available. For more information about our data policy, please see [http://journals.plos.org/plosone/s/data-availability](http://journals.plos.org/plosone/s/data-availability).**

**Upon re-submitting your revised manuscript, please upload your study’s minimal underlying data set as either Supporting Information files or to a stable, public repository and include the relevant URLs, DOIs, or accession numbers within your revised cover letter. For a list of acceptable repositories, please see [http://journals.plos.org/plosone/s/data-availability#loc-recommended-repositories](http://journals.plos.org/plosone/s/data-availability#loc-recommended-repositories). Any potentially identifying patient information must be fully anonymized.**

**Important: If there are ethical or legal restrictions to sharing your data publicly, please explain these restrictions in detail. Please see our guidelines for more information on what we consider unacceptable restrictions to publicly sharing data: [http://journals.plos.org/plosone/s/data-availability#loc-unacceptable-data-access-restrictions](http://journals.plos.org/plosone/s/data-availability#loc-unacceptable-data-access-restrictions). Note that it is not acceptable for the authors to be the sole named individuals responsible for ensuring data access.**

**We will update your Data Availability statement to reflect the information you provide in your cover letter.**

Response: The minimal data sets has been attached as a supporting information file in the revised submission

5. We note that you have stated that you will provide repository information for your data at acceptance. Should your manuscript be accepted for publication, we will hold it until you provide the relevant accession numbers or DOIs necessary to access your data. If you wish to make changes to your Data Availability statement, please describe these changes in your cover letter and we will update your Data Availability statement to reflect the information you provide.

**6. We note that you have referenced (ie. Bewick et al. [5]) which has currently not yet been accepted for publication. Please remove this from your References and amend this to state in the body of your manuscript: (ie “Bewick et al. [Unpublished]”) as detailed online in our guide for authors
[http://journals.plos.org/plosone/s/submission-guidelines#loc-reference-style](http://journals.plos.org/plosone/s/submission-guidelines#loc-reference-style)**

Response: This has been amended to reflect unpublished

**7. We note that Figures 1, 2, 3, 4 and 6 in your submission contain map images which may be copyrighted. All PLOS content is published under the Creative Commons Attribution License (CC BY 4.0), which means that the manuscript, images, and Supporting Information files will be freely available online, and any third party is permitted to access, download, copy, distribute, and use these materials in any way, even commercially, with proper attribution. For these reasons, we cannot publish previously copyrighted maps or satellite images created using proprietary data, such as Google software (Google Maps, Street View, and Earth). For more information, see our copyright guidelines: [http://journals.plos.org/plosone/s/licenses-and-copyright](http://journals.plos.org/plosone/s/licenses-and-copyright).
 
We require you to either (1) present written permission from the copyright holder to publish these figures specifically under the CC BY 4.0 license, or (2) remove the figures from your submission:**

**a. You may seek permission from the original copyright holder of Figures 1, 2, 3, 4 and 6 to publish the content specifically under the CC BY 4.0 license.  
 
We recommend that you contact the original copyright holder with the Content Permission Form ([http://journals.plos.org/plosone/s/file?id=7c09/content-permission-form.pdf](http://journals.plos.org/plosone/s/file?id=7c09/content-permission-form.pdf)) and the following text:
“I request permission for the open-access journal PLOS ONE to publish XXX under the Creative Commons Attribution License (CCAL) CC BY 4.0 ([http://creativecommons.org/licenses/by/4.0/](http://creativecommons.org/licenses/by/4.0/)). Please be aware that this license allows unrestricted use and distribution, even commercially, by third parties. Please reply and provide explicit written permission to publish XXX under a CC BY license and complete the attached form.”
 
Please upload the completed Content Permission Form or other proof of granted permissions as an ""Other"" file with your submission.
 
In the figure caption of the copyrighted figure, please include the following text: “Reprinted from [ref] under a CC BY license, with permission from [name of publisher], original copyright [original copyright year].”
 
b. If you are unable to obtain permission from the original copyright holder to publish these figures under the CC BY 4.0 license or if the copyright holder’s requirements are incompatible with the CC BY 4.0 license, please either i) remove the figure or ii) supply a replacement figure that complies with the CC BY 4.0 license. Please check copyright information on all replacement figures and update the figure caption with source information. If applicable, please specify in the figure caption text when a figure is similar but not identical to the original image and is therefore for illustrative purposes only.
The following resources for replacing copyrighted map figures may be helpful:
 
USGS National Map Viewer (public domain): [http://viewer.nationalmap.gov/viewer/](http://viewer.nationalmap.gov/viewer/)
The Gateway to Astronaut Photography of Earth (public domain): [http://eol.jsc.nasa.gov/sseop/clickmap/](http://eol.jsc.nasa.gov/sseop/clickmap/)
Maps at the CIA (public domain): [https://www.cia.gov/library/publications/the-world-factbook/index.html](https://www.cia.gov/library/publications/the-world-factbook/index.html) and [https://www.cia.gov/library/publications/cia-maps-publications/index.html](https://www.cia.gov/library/publications/cia-maps-publications/index.html)
NASA Earth Observatory (public domain): [http://earthobservatory.nasa.gov/](http://earthobservatory.nasa.gov/)
Landsat: [http://landsat.visibleearth.nasa.gov/](http://landsat.visibleearth.nasa.gov/)
USGS EROS (Earth Resources Observatory and Science (EROS) Center) (public domain): [http://eros.usgs.gov/#](http://eros.usgs.gov/)
Natural Earth (public domain): [http://www.naturalearthdata.com/](http://www.naturalearthdata.com/)**

Response: The maps were originally created by the authors; hence no permission was required. However, a statement indicating this has been added as a footnote to the maps.

Reviewers' comments:

Reviewer's Responses to Questions

**Comments to the Author**

1. Is the manuscript technically sound, and do the data support the conclusions?

The manuscript must describe a technically sound piece of scientific research with data that supports the conclusions. Experiments must have been conducted rigorously, with appropriate controls, replication, and sample sizes. The conclusions must be drawn appropriately based on the data presented.

Reviewer #1: Yes

2. Has the statistical analysis been performed appropriately and rigorously?

Reviewer #1: Yes

3. Have the authors made all data underlying the findings in their manuscript fully available?

The [PLOS Data policy](http://www.plosone.org/static/policies.action#sharing) requires authors to make all data underlying the findings described in their manuscript fully available without restriction, with rare exception (please refer to the Data Availability Statement in the manuscript PDF file). The data should be provided as part of the manuscript or its supporting information, or deposited to a public repository. For example, in addition to summary statistics, the data points behind means, medians and variance measures should be available. If there are restrictions on publicly sharing data—e.g. participant privacy or use of data from a third party—those must be specified.

Reviewer #1: No

4. Is the manuscript presented in an intelligible fashion and written in standard English?

PLOS ONE does not copyedit accepted manuscripts, so the language in submitted articles must be clear, correct, and unambiguous. Any typographical or grammatical errors should be corrected at revision, so please note any specific errors here.

Reviewer #1: Yes

5. Review Comments to the Author

Please use the space provided to explain your answers to the questions above. You may also include additional comments for the author, including concerns about dual publication, research ethics, or publication ethics. (Please upload your review as an attachment if it exceeds 20,000 characters)

**Reviewer #1: I read the manuscript PONE-D-22-16005 entitled "Geospatial distribution and predictive modeling of onchocerciasis in Ogun State, Nigeria" with interest. In this study, the authors obtained georeferenced onchocerciasis infection data from a cross-sectional survey collected between March and July 2015 from locations in Ogun State, Nigeria. However, it could have been a very interesting work if communicated earlier, as the MDA interventions in the past seven years might have changed the epidemiological situation of the disease in the area. Though it is possibly losing its merit, this will provide useful information for identifying priority areas for delivery of intervention and eventually contribute to the elimination of onchocerciais in the study area.
The authors followed appropriate statistical methods and modelling to analyse the data. My comments on the manuscript are on the use of abbreviations and punctuation throughout the manuscript. There are many small errors in punctuation and improper use of abbreviations throughout the manuscript. For example, in the abstract portion, the abbreviation "LGA" should be written in its long form as it appears first, then in its abbreviated form. The same is true for the phrases "mass drug administration", "community-directed treatment with ivermectin", "Onchocerca volvulus" etc.. whereas WHO, GIS, RS, WHO-NTD, RDT, mf, AOR etc ... are written in abbreviated form in their first appearance in the manuscript. These and other terms in the manuscript have to be critically looked and corrected. If the manuscript is to be accepted, there may be a need for a language check by a professional editor for errors in punctuation in the manuscript.
Moreover, the authors need to follow the PLOSONE authors’ guide to format the manuscript, especially the ethical clearance and permission subsection, which needs to be placed before the result section of the manuscript.**

Response: All punctuation errors have been addressed and abbreviations have been spelt out during first mention.

6. PLOS authors have the option to publish the peer review history of their article ([what does this mean?](https://journals.plos.org/plosone/s/editorial-and-peer-review-process#loc-peer-review-history)). If published, this will include your full peer review and any attached files.


If you choose “no”, your identity will remain anonymous but your review may still be made public.


**Do you want your identity to be public for this peer review?** For information about this choice, including consent withdrawal, please see our [Privacy Policy](https://www.plos.org/privacy-policy).

Reviewer #1: No

[NOTE: If reviewer comments were submitted as an attachment file, they will be attached to this email and accessible via the submission site. Please log into your account, locate the manuscript record, and check for the action link "View Attachments". If this link does not appear, there are no attachment files.]

While revising your submission, please upload your figure files to the Preflight Analysis and Conversion Engine (PACE) digital diagnostic tool, [https://pacev2.apexcovantage.com/](https://pacev2.apexcovantage.com/). PACE helps ensure that figures meet PLOS requirements. To use PACE, you must first register as a user. Registration is free. Then, login and navigate to the UPLOAD tab, where you will find detailed instructions on how to use the tool. If you encounter any issues or have any questions when using PACE, please email PLOS at [figures@plos.org](mailto:figures@plos.org). Please note that Supporting Information files do not need this step.
