# Supplementary material for: Geospatial distribution and predictive modeling of onchocerciasis in Ogun State, Nigeria
Source: PLoS One. 2023 Mar 1;18(3):e0281624. doi: 10.1371/journal.pone.0281624 (PMC9977021; doi:10.1371/journal.pone.0281624)
Supplement: Attachment — Submitted filename: Comments_PONE_D_22_16005_R1.docx [file pone.0281624.s003.docx]

**Comments**

There are improvements from the previous version of the manuscript. However, the comments I raised in the previous review are not fully addressed. As mentioned earlier, most of the problem in the current version of the manuscript is with the use of abbreviations. There is improper use of abbreviations throughout the manuscript. If you started using the abbreviated version of one word or phrase, you have to use the abbreviated form in the next part of the manuscript. Some of the errors in the manuscript are mentioned here below. Moreover, you have to critically look the manuscript for other similar errors and correct it accordingly.

Line 31: The abbreviation "LGA" should be written in its long form as it appears first, then in its abbreviated form.

Line 33: Please write onchocerca volvulus in its abbreviated form, i.e., "O. volvulus".

Line 55: Please write onchocerca volvulus in its abbreviated form, i.e., "O. volvulus" . Similar comments to lines 76, 142, 143, 144, 214, 252, 254, 261, 267, 268, and 397.

 Lines 66 and 67: The phrase "community-directed treatment with ivermectin", should be written in abbreviated form, as it had already been abbreviated in line 61 of the manuscript.

 Line 68 and 69: The phrase "mass drug administration" should be written as "MDA" as you have already started using the abbreviated form of it in line 58 of the manuscript. Also, similar comment to line 407 of the manuscript.

Line 69: Write the abbreviated version of microfilaria in parantheses i.e. microfilaria. Also, use the abbreviated form of microfilaria in lines 97 and 127 of the manuscript.

Line 111: Use "LGAs" instead of "Local Government Areas".

Line 118 and 119: Use the abbreviated form of Rapid Diagnostic Test i.e. "RDT".

Line 150: Please use "19" instead of "nineteen".

Line 262: Abbreviate the "Crude Odd ratio" as "COR", then, start to use this abbreviation following it.

Line 219: Please use "two" instead of "2".

Line 221: Add "percent" next to "zero" i.e. zero percent prevalence.

Lines 267 and 269: replace "y" with "years".

Line 299: Please replace "0" with "zero percent".

Line 317: Please use the abbreviated version for "Receiver Operating Characteristics".

Line 319: Please use the abbreviated version of "Areas under curve". Similar comment to lines 323 and 324.

Line 394: Replace "8" with "eight".

Line 404: Please replace "it’s" with "it is".

Line 426: Please use the abbreviated version of the species name. i.e. S. damnosum s.l.

Line 461 and 462: Please use the abbreviated form of "maximum entropy Modelling" only .i.e. "MaxEnt".
