# Supplementary material for: Geospatial distribution and predictive modeling of onchocerciasis in Ogun State, Nigeria
Source: PLoS One. 2023 Mar 1;18(3):e0281624. doi: 10.1371/journal.pone.0281624 (PMC9977021; doi:10.1371/journal.pone.0281624)
Supplement: Attachment — Submitted filename: Reviewers response D_22_16005_R1.docx [file pone.0281624.s004.docx]

**Response to reviewers comment**

Line 31: The abbreviation "LGA" should be written in its long form as it appears first, then in its abbreviated form.

Response: The abbreviation has been re-written in the long form as Local Government Areas

Line 33: Please write onchocerca volvulus in its abbreviated form, i.e., "O. volvulus".

Response: This has been changed in all places it appeared as *O. volvulus* to *O. volvulus*

Line 55: Please write onchocerca volvulus in its abbreviated form, i.e., "O. volvulus" . Similar comments to lines 76, 142, 143, 144, 214, 252, 254, 261, 267, 268, and 397.

Response: This has been changed in all places it appeared as *O. volvulus* to *O. volvulus*

 Lines 66 and 67: The phrase "community-directed treatment with ivermectin", should be written in abbreviated form, as it had already been abbreviated in line 61 of the manuscript.

Response: The phrase has been written in the abbreviated form

 Line 68 and 69: The phrase "mass drug administration" should be written as "MDA" as you have already started using the abbreviated form of it in line 58 of the manuscript. Also, similar comment to line 407 of the manuscript.

Response: The phrase has been written in the abbreviated form

Line 69: Write the abbreviated version of microfilaria in parantheses i.e. microfilaria. Also, use the abbreviated form of microfilaria in lines 97 and 127 of the manuscript.

Response: The abbreviated form of microfilaria has been written in parenthesis

Line 111: Use "LGAs" instead of "Local Government Areas".

Response: Local Government Areas has been re-written as LGAs

Line 118 and 119: Use the abbreviated form of Rapid Diagnostic Test i.e. "RDT".

Response: The abbreviated form of Rapid Diagnostic Test has been used

Line 150: Please use "19" instead of "nineteen".

Response: Line 150 has been corrected to reflect 19

Line 262: Abbreviate the "Crude Odd ratio" as "COR", then, start to use this abbreviation following it.

Response: Crude Odd ratio has been abbreviated to reflect as "COR”

Line 219: Please use "two" instead of "2".

Response: Line 219 has been corrected to reflect two

Line 221: Add "percent" next to "zero" i.e. zero percent prevalence.

Response: Percent has been added to zero

Lines 267 and 269: replace "y" with "years".

Response: Years has been used to replace y in all places it appears

Line 299: Please replace "0" with "zero percent".

Response: O has been replaced with Zero percent

Line 317: Please use the abbreviated version for "Receiver Operating Characteristics".

Response: The abbreviated version for "Receiver Operating Characteristics" has been replaced with ROC

Line 319: Please use the abbreviated version of "Areas under curve". Similar comment to lines 323 and 324.

Response: The abbreviated version for " Areas under curve " has been replaced with AUC.

Line 394: Replace "8" with "eight".

Response: 8 has been replaced with eight

Line 404: Please replace "it’s" with "it is".

Response: It’s has been replaced with it is

Line 426: Please use the abbreviated version of the species name. i.e. S. damnosum s.l.

Response: The abbreviated version of the species name has been used

Line 461 and 462: Please use the abbreviated form of "maximum entropy Modelling" only .i.e. "MaxEnt".

Response: MaxEnt has been replaced with maximum entropy Modelling
